# Prevalence of chronic viral hepatitis B, D among Mongol migrants in Sweden compared to a sex- and age-matched native Mongol cohort

**DOI:** 10.1186/s12879-025-12506-w

**Published:** 2026-01-16

**Authors:** Delgersaikhan Zulkhuu, Habiba Kamal, Sanjaasuren Enkhtaivan, Ochirmaa Narantsogt, Ganbolor Jargalsaikhan, Munguntsetseg Batkhuu, Byambasuren Ochirsum, Marcus Ahl, Karin Lindahl, Michael Ingre, Natalie Stiglund, Alexandros Petropoulos, Gustaf Sandh, Niklas K. Björkström, Sonja Saario, Susanne Cederberg, Annika Olsson, Anna Wange Baye, Ihssan Derdabi, Desideria Georgos, Andreas S. Bungert, Nara Bungert Dashdorj, N. D. Dashdorj Onom, Soo Aleman

**Affiliations:** 1 The Liver Center, Ulaanbaatar, Mongolia; 2 Onom Foundation, Ulaanbaatar, Mongolia; 3 Department of Infectious Diseases, Karolinska University Hospital, Stockholm, Sweden; 4 Department of Medicine Huddinge, Karolinska Institutet, Stockholm, Sweden; 5 D-SOLVE Consortium, an EU Horizon Europe Funded Project, Hanover, Germany; 6 Centre for Bioinformatics and Biostatistics, Karolinska Institutet, Stockholm, Sweden; 7 Department of Clinical Microbiology, Medical Diagnostics Karolinska, Karolinska University Hospital, Stockholm, Sweden; 8 Center for Infectious Medicine, Department of Medicine Huddinge, Karolinska Institutet, Karolinska University Hospital, Stockholm, Sweden; 9 Department of Microbiology, Tumor and Cell Biology, Karolinska Institutet, Stockholm, Sweden; 10 Division of Clinical Microbiology, Department of Laboratory Medicine, Karolinska Institutet, Stockholm, Sweden; 11 Onom Institute, San Jose, CA USA

**Keywords:** Hepatitis D, Mongolia, Screening, HBV, HCV, Prevalence, Migrants, Health disparity

## Abstract

**Background & aims:**

Chronic hepatitis B/D is the most severe form of chronic viral hepatitis, yet public awareness of its burden remains low. This study examined the prevalence of viral hepatitis B and D among Mongol migrants in Sweden and facilitated linkage to care.

**Methods:**

We conducted a viral hepatitis screening event on January 28th -29th, 2023 at Karolinska University Hospital, Stockholm, for individuals of Mongol descent. Consenting participants underwent blood testing and liver stiffness measurements. The prevalence of viral hepatitis was compared with an age- and sex-matched general population cohort in Mongolia.

**Results:**

We screened 850 adult Mongols (mean age (SD) 43.2 ± 8.6 years; 61.9% women). The prevalence of HBsAg+, anti-HDV+, and HDV-RNA + were 4.4%, 2.1%, and 1.3%, respectively. The corresponding prevalences in the matched native Mongol cohort were significantly higher at 9.8%, 5.1%, and 3.7%. Among HBsAg + individuals, 48.6% were anti-HDV+; 61.1% of anti-HDV + individuals had detectable HDV-RNA. Prior HBV infection was identified in 62.9% of participants, 8.7% had evidence of vaccination, and 22.8% were susceptible to HBV infection. Overall, 58 (6.8%) individuals with chronic viral hepatitis were linked to care. Among HBsAg + or anti-HBc + individuals, only 54.1% and 65.8% were aware of their current or past HBV infection, respectively. Anti-HCV + prevalence was 18.6%; 13.3% had detectable HCV-RNA, indicating chronic infection.

**Conclusion:**

Mongol migrants had lower prevalences of hepatitis B and D than the general population in Mongolia, but higher than in the Swedish general population. Increased screening efforts are needed to enhance awareness, improve diagnosis and linkage to care for populations at higher risk of chronic viral hepatitis in Sweden and Europe.

**Supplementary Information:**

The online version contains supplementary material available at 10.1186/s12879-025-12506-w.

## Introduction

Screening for viral hepatitis is an essential first step in the continuum process of prevention, diagnosis, and care for chronic viral hepatitis and related complications [1]. The burden of chronic hepatitis B, C, and D remains high in Asia, Eastern Europe, the Middle East, and Sub-Saharan Africa [1]. Consequently, the ten countries with the highest age-standardized incidence rates of liver cancer and related mortality are endemic with chronic viral hepatitis B, C, and D [2].

Migrants and other vulnerable groups, such as people who inject drugs and incarcerated individuals, have a higher prevalence of chronic viral hepatitis B, C and D and are at increased risk of delayed or missed linkage to care [3, 4]. Additional barriers for migrants may include lower disease awareness, language barriers, stigma, and socioeconomic challenges in host countries [5]. These barriers vary across countries depending on the specific context and national strategies for addressing viral hepatitis [3, 5].

Chronic hepatitis D virus (HDV) infection is the most severe form of viral hepatitis. HDV requires hepatitis B virus (HBV) for infection and causes accelerated progression to cirrhosis and end-stage liver disease. Estimates of the global population chronically infected with HDV vary widely, ranging from 10 to 70 million individuals [6, 7].

In Sweden, the prevalence of HBV and hepatitis C virus (HCV) is low, estimated at approximately 30,000 (~ 0.3%) and 20,000 (~ 0.2%) individuals, respectively [8, 9], whereas approximately 1,000 individuals (~ 0.01%) live with chronic HDV infection [10]. Sweden has demonstrated progress towards eliminating viral hepatitis, with a 40% decrease in new HBV notifications during 2015–2018 [8].

Similar to other low-endemic European settings, most individuals living with HBV in Sweden were infected perinatally, and originate from highly endemic countries, while the majority of individuals with HCV were infected domestically [8].

Mongolia is heavily burdened by viral hepatitis and liver cancer [11, 12]. Despite the introduction of neonatal HBV vaccination in 1991, the prevalence of HBV and HDV infection remains high at 11.1%, 6.6%, respectively, and 8.5% are infected with HCV [11, 13]. Previous screening initiatives of Mongol migrants in the United States revealed a 9.0% prevalence of HCV and HBV infection; among those with HBV, 41.2% were anti-HDV positive [14]. In contrast, a recent study in Spain reported lower estimates of 5.8% for HCV and 3.6% for HBV [15].

No prior screening for HBV and HDV infections among Mongol migrants has been conducted in Sweden. Therefore, this study aimed to assess the prevalence of viral hepatitis B and D, identify infected individuals, and facilitate linkage to care for those in need within this population.

## Methods

### Study design and participants

We conducted a cross-sectional, population-based screening of Mongol migrants living in Sweden. Participants were recruited through social media platforms using culturally and linguistically tailored invitations.

This screening event was a collaborative initiative between the Infectious Diseases Clinic at Karolinska University Hospital (KUH), a secondary referral center covering the Southern Stockholm region; the Onom Foundation; a family foundation committed to reducing premature mortality among Mongols in and outside Mongolia; the Liver Center; a center of excellence for screening, diagnosis, treatment and research of liver diseases in Ulaanbaatar, Mongolia; the D-Solve Consortium; a European network coordinated by Hannover Medical School (MHH) and the Centre for Individualized Infection Medicine (CIIM) dedicated to screen and study HDV infection in large multicenter cohorts [16]; and the Onom Institute, a non-profit private research institute in the United States.

Applicants completed a questionnaire covering demographic parameters and other relevant information (Supplementary Table 1).

On the screening days (28–29 January 2023), Swedish and Mongol healthcare personnel delivered lectures on viral hepatitis, and informational brochures were distributed. All participants signed an informed consent form in Mongolian that explained the procedures included in the screening. Nurses collected venous blood samples for virological and laboratory testing. Liver stiffness (LS) and controlled attenuation parameter (CAP) values were evaluated using transient elastography in all participants. We considered LS measurements reliable when the success rate was ≥ 90% and the interquartile range (IQR) was ≤ 30% [17]. Participants received their test results 2–3 weeks after screening.

### General population cohort in Ulaanbaatar, Mongolia

We selected an age- and sex-matched cohort (1:1) from 2,600 individuals who participated in the General Population STEPS Survey conducted in Ulaanbaatar, Mongolia from December 2017 through January 2018 [18]. We randomly selected 413 participants from this cohort, whose serum samples were screened for viral hepatitis B and D infection.

### Definitions and co-variates

As information on prior HBV vaccination status was not collected, we subgrouped individuals based on serological evidence as follows: those with prior HBV infection (HBsAg-, anti-HBc+); those with evidence of prior HBV vaccination (HBsAg-, anti-HBc- and anti-HBs+); those susceptible to infection (HBsAg-, anti-HBc- and anti-HBs-); and those with chronic HBV infection (HBsAg+, anti-HBc+) [19–21].

We also screened for HCV seroprevalence. Individuals with positive HCV antibody were tested for HCV-RNA replication. Those with chronic infection were offered antiviral therapies with follow-up.

We classified LS values as follows: <7.5 kPa corresponded to liver fibrosis stages F0-F1 (no to early fibrosis), 7.5–12.4 kPa to F2 - F3 (moderate to advanced fibrosis), and ≥ 12.5 kPa to F4 (cirrhosis) [17]. We informed individuals with LS between 7.5 and 12.4 kPa about possible causes of elevated LS, such as diet or alcohol overconsumption, and referred them to their primary care clinics for follow-up. Individuals with LS ≥ 12.5 kPa were subjected to further examination. CAP scores between 291 and 400 decibels per meter (dB/m) indicated severe steatosis or S3 (> 2/3 steatosis of the liver) [22].

Body mass index (BMI) was calculated as weight (kilogram)/ height^2^ (meters). Following recommendations for Asian populations, we classified BMI < 23.0 kg/m² as normal range, 23.0–27.4 kg/m² as overweight, and ≥ 27.5 kg/m² as obese [23]. The permanent identification number (PIN) is obtained by Swedish residents at birth or upon immigration to Sweden [8]. This number serves as a unique identifier throughout an individual’s life and is linked to all national Swedish registers. Individuals lacking a PIN may obtain temporary registration numbers at the local level when accessing healthcare services [8]. We calculated the length of stay in Sweden by subtracting age at immigration from age at the screening event.

### Study outcomes

Our primary outcome was to assess the prevalence of HBV, and HDV infections. Other parameters of interest included HCV, and HIV seroprevalences, serological evidence of prior HBV infection, prior HBV vaccination, susceptibility to HBV, and self-awareness of viral hepatitis infection and of an infected family member.

The secondary outcomes included the frequency of linkage to care, such as the number of patients who initiated anti-HBV or novel anti-HDV therapies or received direct-acting antiviral (DAA) anti-HCV therapy, as well as those who initiated hepatocellular carcinoma (HCC) surveillance. A comparison of the prevalence of HBV and HDV in a matched general Mongol population cohort in Ulaanbaatar, Mongolia was also performed.

### Analyses of virological parameters

At KUH laboratory, qualitative detection of HBsAg, anti-HCV, anti-HIV/HIVAg, and anti-HBc was performed on a Cobas 8000 e801 instrument (ECLIA; Roche Diagnostics GmbH, Germany) using the Elecsys HBsAg II, Elecsys anti-HCV II, Elecsys HIV Duo, and Elecsys anti-HBc II assays (Roche Diagnostics GmbH, Germany). Quantitative detection of anti-HBs and HBsAg was performed using Elecsys anti-HBs II and Elecsys HBsAg II quant II on the Cobas 8000 e801 instrument (Roche Diagnostics GmbH, Germany). The qualitative detection of anti-HDV was performed using the Liaison XL murex Anti-HDV (CLIA) assay on the LIAISON XL instrument (DiaSorin S.p.A., Italy). HDV-RNA samples were quantified by quantitative real-time PCR (qPCR) using the RoboGene HDV-RNA quantification kit 2.0 (AnalytikJena AG, Germany), with a lower limit of detection (LLoD) of 6 IU/mL, following nucleic acid extraction using the MagNA Pure 96 DNA and Viral NA Small Volume Kit on the MagNA Pure 96 instrument (Roche Diagnostics GmbH, Germany). HBV-DNA and HCV-RNA levels were quantified using the Cobas HBV, and Cobas HCV Quantitative nucleic acid tests on Cobas 6800/8800 (Roche Diagnostics GmbH, Germany).

At the Clinical and Molecular Diagnostics Laboratory of the Liver Center, HBsAg was qualitatively tested using rapid diagnostic test (CTK Biotech Inc., USA), while anti-HDV was tested using ELISA (Wantai Co. Ltd., PRC). Quantitative HBsAg was estimated using HISCL-5000 fully automated chemiluminescence analyzer (Sysmex Corporation, Japan). HDV-RNA was quantified using real-time reverse transcriptase polymerase chain reaction with a limit of detection (LOD) of 10 IU/mL (Bioactiva Diagnostica, Germany; Bio-Rad, USA).

### Statistical analysis

Continuous variables were presented as mean ± standard deviation (SD) when normally distributed, and as median (interquartile range, IQR) when skewed. We used Student’s t-test and Mann-Whitney tests for comparison of normal and skewed continuous variables, respectively. Categorical variables were presented as frequencies (numbers) and proportions and we compared them using the Chi-Square test or Fisher’s exact test, as appropriate. We performed univariable and multivariable logistic regression analyses generating odds ratio with 95% confidence intervals (CI) to test the association of elevated LS ≥ 7.5 kPa with the following variables: age at the event, sex, BMI, duration of stay in Sweden, positive family history of HBV infection, and positive serological results for viral hepatitis. We considered statistical significance at p-value < 0.05. We executed all analyses using R version 4.2.2 and IBM-SPSS, version 27. Data handling and figures were created using Microsoft excel 365. Data analysis was performed in June 2024.

### Power calculation for the native cohort

We matched a 1:1 age- and sex-matched cohort from the general population in Ulaanbaatar, Mongolia to the Stockholm screened participants (*n* = 850). This general population cohort included 2,600 individuals who participated in the Ulaanbaatar STEPS Survey [18]. Assuming the prevalence of HDV in the general population at 5.2%, 413 individuals were needed to achieve 80% power to detect a difference. We randomly selected this number from the matched cohort.

## Results

### Study participants

**Fig. 1:**
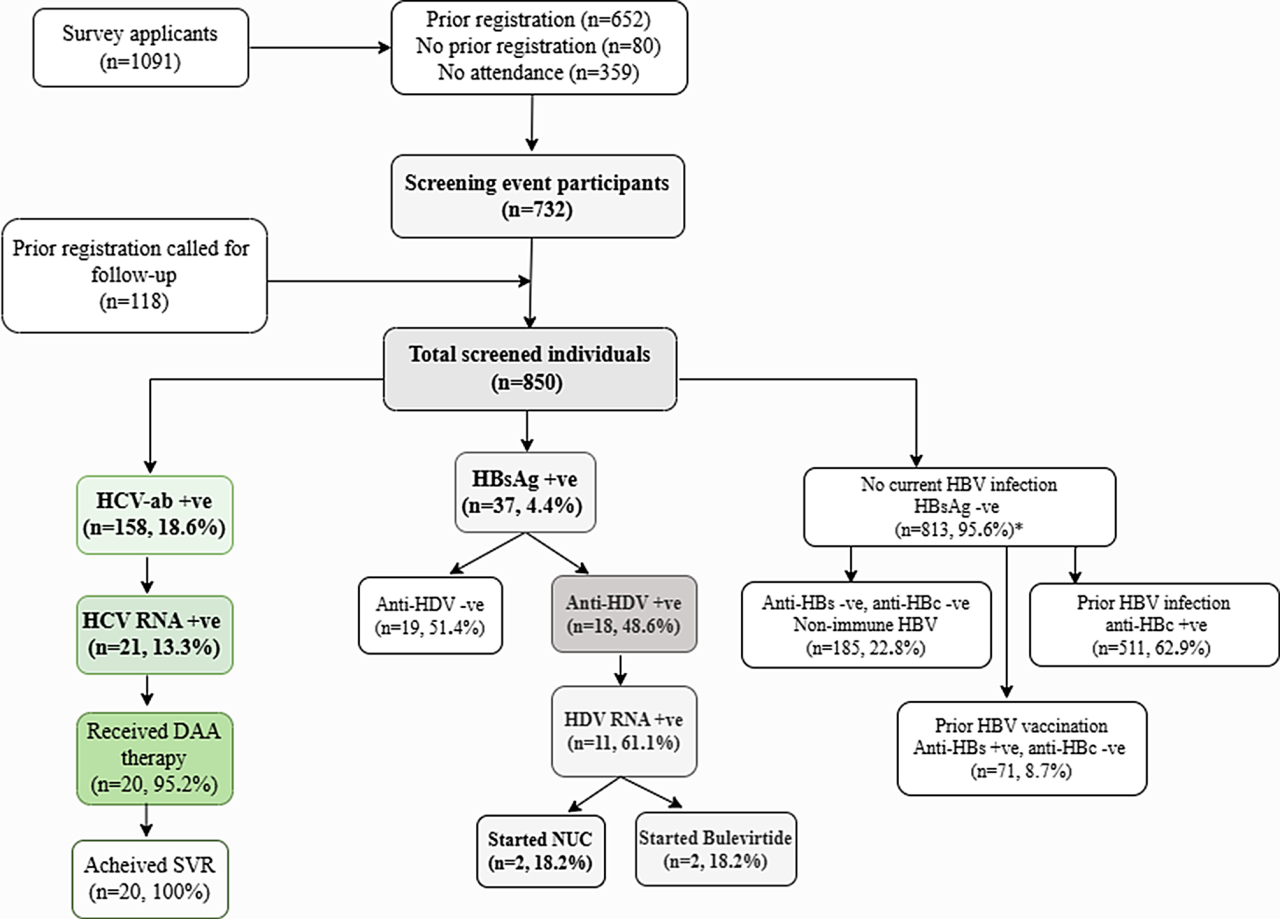
Flowchart of study participants. Abbreviations: HBV=hepatitis B virus; HBsAg: hepatitis B surface antigen, HDV= hepatitis D virus; anti-HDV=hepatitis D virus antibody; HDV RNA= hepatitis D virus ribonucleic acid; HCV-ab= hepatitis C virus antibody; HCV RNA= hepatitis C virus ribonucleic acid; anti-HBc= anti-hepatitis B core; anti-HBs=hepatitis B surface antibody; DAA= direct acting antiviral; SVR=sustained virological response defined as undetected HCV RNA ≥ 12 weeks post treatment end; NUC= nucleot(s)ides analogues;* 46 patients missing anti-HBs

Figure 1 shows the study flowchart. Table 1 presents the baseline characteristics of participants.

**Table 1:** Baseline characteristics of participants in the screening event, sub-grouped by sex

Parameters**	All, *n* (%)	Men, *n* (%)	Women, *n* (%)	*p*-value
Number (%) unless stated otherwise	850 (100)	324 (38.1)	526 (61.9)	< 0.001
Year of migration, median (IQR)	2015 (2011–2018)	2015 (2011–2018)	2015 (2011–2018)	0.47
Mean age at migration, mean (SD, min-max)	34.4 (8.7, 6.0–68.0)	33.3 (8.4, 9.1–60.2)	35.2 (8.9, 6–68)	0.003
Mean age at the event, years (SD, min-max)	43.2 (8.6, 18.0-75.8)	42.0 (8.2, 19.2–64.9)	43.9 (8.8, 18.0-75.8)	0.002
Presence of a permanent Swedish personal identification number	675 (79.4)	269 (83.0)	406 (77.2)	0.04
**Prior HBV screening**,				
Yes	164 (19.3)	59 (18.2)	105 (20.0)	0.52
No	564 (66.4)	225 (69.4)	339 (64.4)	
Do not know	122 (14.4)	40 (12.3)	82 (15.6)	
**Have you been diagnosed with HBV infection**	*726*	*278*	*448*	
Yes	59 (8.1)	24 (8.6)	35 (7.8)	0.47
No	493 (67.9)	196 (70.5)	297 (66.3)	
Do not know	174 (24.0)	58 (20.9)	116 (25.9)	
**BMI**,** kg/m**^**2**^	*841*	*320*	*521*	
Mean (SD)	26.1 (4.3)	26.9 (3.7)	25.7 (4.6)	< 0.001
<23	183 (21.8)	43 (13.4)	140 (26.9)	< 0.001
23-27.5	366 (43.5)	140 (43.8)	226 (43.4)	0.18
≥27.5	292 (34.7)	137 (42.8)	155 (29.8)	< 0.001
**HBV**				
HBsAg+	37 (4.4)	16 (4.9)	21 (4.0)	0.52
HBV-DNA log_10_ (IU/ml), median (IQR)	2.4 (1.2–2.9)	2.3 (1.2–3.8)	2.4 (1.0–2.7)	0.27
HBsAg log_10_ (IU/ml), median (IQR)	2.6 (1.9–3.7)	3.3 (2.1–3.7)	2.3 (1.1–3.7)	0.06
HBsAg-	*813*	*308*	*507*	
Prior HBV infection (HBsAg-, anti-HBc+)	511 (62.9)	204 (66.2)	307 (60.8)	0.14
Vaccinated (HBsAg-, anti-HBc-, anti-HBs+)	71 (8.7)	23 (7.5)	48 (9.5)	0.93
Susceptible to HBV (HBsAg-, anti-HBc-, anti-HBs-)	185 (22.8)	61 (19.8)	124 (24.6)	0.15
Missed anti-HBs	46 (5.7)	20 (6.5)	26 (5.1)	0.42
**Knowledge about current infection among HBsAg+**	*37*	*16*	*21*	
Yes	20 (54.1)	9 (56.3)	11 (52.4)	0.19
No	8 (21.6)	4 (25.0)	4 (19.0)	0.70
Do not know	9 (24.3)	3 (18.8)	6 (28.6)	0.20
**HDV**				
Anti-HDV+	18 (2.1)	5 (1.5)	13 (2.5)	0.36
HDV-RNA+	11 (1.3)	4 (1.2)	7 (1.3)	0.31
HDV-RNA log_10_ IU/ml, median (IQR)	4.4 (1.9–4.8)	4.7 (4.2–5.7)	4.0 (1.7–4.8)	0.12
**HCV**				
Anti-HCV+	158 (18.6)	59 (18.2)	99 (18.8)	0.84
HCV-RNA+	21 (2.5)	12 (3.7)	9 (1.7)	0.07
HCV-RNA log_10_ IU/ml, median (IQR)	6.0 (5.5–6.6)	6.2 (5.5–6.8)	6.0 (5.1–6.5)	0.23
**Liver stiffness values**	*819*	*315*	*504*	
LS, median (IQR), kPa	4.5 (3.7–5.6)	4.8 (4.1–5.9)	4.3 (3.5–5.4)	0.002
CAP score	***338***	*134*	*204*	
median (IQR) dB/m	236.5 (202.0-277.3)	264.0 (216.8-290.5)	226.0 (193.3-270.8)	< 0.001
S3 ≥ 290	76 (22.5)	42 (31.3)	34 (16.7)	0.001

Number of participants with available data is given in italic. Abbreviations: n = number; anti-HDV = hepatitis D antibody; HDV-RNA = hepatitis D virus ribonucleic acid; HBV = hepatitis B virus; anti-HCV + = hepatitis C antibody; SD = standard deviation; IQR = 25th -75th interquartile, 25th and 75th percentiles are displayed; BMI = body mass index calculated as body weight in kilogram/(height in meters)^2^; LSM = liver stiffness measurements; kPa = kilopascal; CAP = controlled attenuated parameter score measured in decibels per meter (dB/m); S3 = CAP score 290 to 400 dB/m translating to > 2/3 of the liver has steatosis

**Fig. 2:**
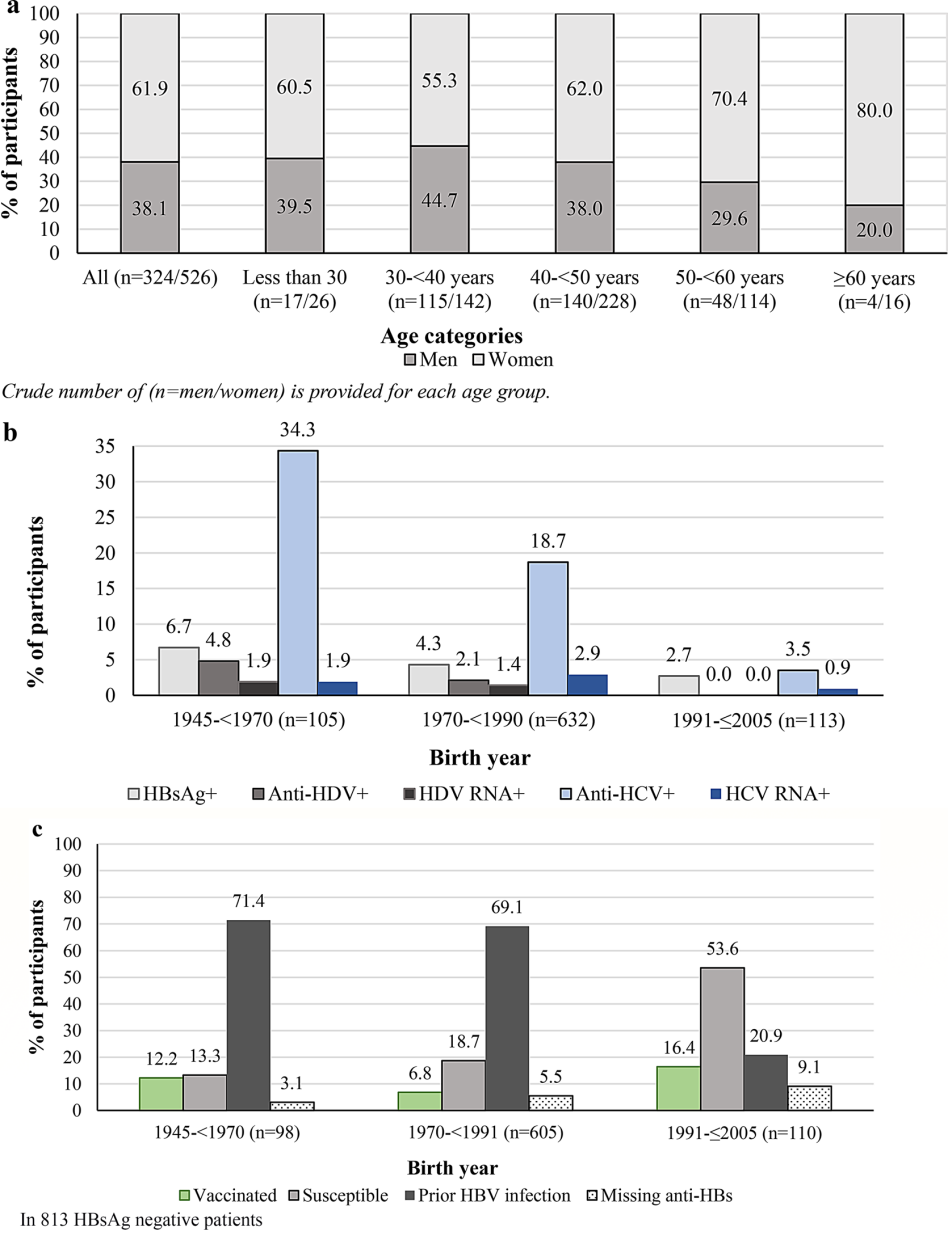
**a**) Distribution of participants by sex and age categories, **b**) Prevalence of viral hepatitis by year of birth, **c**) Prior HBV exposure or vaccination by year of birth

Of 1,091 survey applicants, the final cohort comprised 850 individuals: 732 were initial attendees, and 118 persons attended during January-May 2024 through follow-up calls. Women constituted (61.9%, 526/850) of participants, women outnumbered men across all age categories (Fig. 2a). Women were older than men at migration (mean age 35.2 vs. 33.3 years, *p* = 0.003) and at the screening event (mean age 43.9 vs. 42.0 years, *p* = 0.002). Overall, 79.4% (675/850) of study participants possessed a PIN, with a lower frequency in women (77.2%, 406/526) compared with men (83.0%, 269/324). Nearly all participants (98.6%, 838/850) reported living in Stockholm.

### Study outcome (Table 1)

Among all participants, the prevalence of HBsAg+, anti-HDV+, anti-HCV+, and anti-HIV/HIV Ag + was 4.4% (37/850), 2.1% (18/850), 18.6% (158/850), and 0%, respectively. Among HBsAg + individuals, 48.6% (18/37) were anti-HDV+, and 61.1% (11/18) of anti-HDV + had detectable HDV-RNA at a median of 4.4 log_10_ IU/mL (IQR1.9-4.8). Among individuals with anti-HCV+, 13.3% (21/158) had HCV-RNA replication with genotype 1b (one patient was excluded after a borderline value tested negative upon repeat testing).

Across birth cohorts, the prevalence of anti-HCV + significantly declined from 34.3% (36/105) in the 1945-<1970 subgroup to 3.5% (4/113) in 1991–2005 subgroup, demonstrating a birth cohort effect. We observed a similar decline for HBsAg + and anti-HDV + across these birth cohorts, with the lowest prevalences found in the 1991–2005 birth cohort (Fig. 2b) (*p* < 0.001).

Excluding patients with confirmed HBV infection, nearly two-thirds 62.9% (511/813) of participants had resolved HBV infection, 8.7% (71/813) showed evidence of prior vaccination, and 22.8% (185/813) were susceptible to HBV infection. Younger participants (born 1991-≤2005) had a higher prevalence of individuals with detected anti-HBs+ (suggesting prior vaccination) at 16.4% (18/110), were the least likely to have prior HBV infection at 20.9% (23/110), and 53.6% (59/110) were susceptible to infection (all *p* < 0.001) (Fig. 2c). Only one individual (0.12%) had HBsAg+, anti-HDV+, and anti-HCV+, and no participants tested positive for HIV.

### Characteristics of participants with prior or current HBV, HDV and HCV infection (Table 2)

**Table 2:** Baseline characteristics of participants according to detected viral hepatitis

Parameters, *n* (%) unless stated otherwise	HBsAg+, anti-HDV-	HBsAg+, anti-HDV+	Anti-HCV+	*p*-value
Number, % of 850 participants	19, 2.3%	18, 2.1%	158, 18.6%	< 0.001
Sex, women	8 (42.1)	13 (72.2)	99 (62.7)	< 0.001
Mean age at the event, years, (SD)	42.2 (9.1)	47.6 (6.7)	47.1 (8.2)	0.039
Year of arrival to Sweden, median (IQR) year	2015 (2010–2018)	2017 (2012–2021)	2015 (2011–2018)	0.092
Presence of a permanent Swedish personal number	13 (68.4)	12 (66.7)	122 (77.2)	< 0.001
Prior test to HBV	10 (52.6)	10 (55.6)	30 (19.0)	< 0.001
**Awareness of current viral hepatitis infection**				
Yes	11 (57.8)	9 (50.0)	16 (10.2)	0.011
No	4 (21.1)	4 (22.2)	74 (46.5)	< 0.001
I do not know	4 (21.1)	5 (27.8)	68 (43.3)	< 0.001
**Awareness of a family or household member infected with HBV**	*11*	*9*	*82*	
Yes	2 (18.2)	2 (22.2)	10 (12.2)	0.008
No	6 (54.5)	5 (55.6)	45 (54.9)	0.87
I do not know	3 (27.3)	2 (22.2)	27 (32.9)	< 0.001
**BMI**	*19*	*18*	*156*	
Mean (SD), kg/m^2^	26.8 (3.5)	27.5 (9.9)	26.8 (4.4)	0.725
<23 kg/m^2^	1 (5.3)	2 (11.1)	25 (16.0)	< 0.001
23–27.5 kg/m^2^	12 (63.2)	13 (72.2)	71 (45.5)	< 0.001
≥27.5 kg/m^2^	6 (31.6)	3 (16.7)	60 (38.5)	< 0.001
**Virological markers**				
Anti-HCV+	0	1 (5.6)	158 (100)	< 0.001
HCV-RNA+	-	0	21 (13.4)^	
HDV-RNA +	-	11 (61.1)	0	
**LS**	*19*	*18*	*150*	
Median (IQR), kPa	4.5 (3.7–5.5)	6.5 (4.0–8.2)	4.7 (3.8–5.9)	< 0.001
LS ≥ 9.0 kPa	1 (5.3)	3 (16.7)	11 (7.3)	< 0.001
LS ≥ 12.5 kPa	0	1 (5.6)	3 (2.0)	0.014
**CAP**	*13*	*11*	*64*	
median (IQR) db/m	239.0 (208.5–308.0)	223.0 (193.0- 271.0)	229.5 (202.0-271.0)	0.04
CAP ≥ 290 db/m	4 (30.8)	1 (9.1)	10 (15.6)	< 0.001

Number of participants with available data is given in italic. Abbreviations: anti-HDV = hepatitis D antibody; HDV-RNA = hepatitis D virus ribonucleic acid; HBV = hepatitis B virus; BMI = body mass index; anti-HCV = hepatitis C antibody; kPa = kilopascal; n = number; IQR = 25th -75th interquartile; LS = liver stiffness; CAP = controlled attenuated parameter score measured in decibels per meter (dB/m); S3 = CAP score 290 to 400 dB/m translating to > 2/3 of the liver has steatosis. N.B: one patient had repeated HCV-RNA determined negative thereafter

Individuals with anti-HDV + and anti-HCV + were significantly older than those with HBsAg+ (mono-infection), with similar years of migration to Sweden but slightly earlier for those with anti-HCV+. Women constituted the majority 72.2% (13/18) of individuals with anti-HDV + compared with 42.1% (8/19) of HBV mono-infection and 62.7% (99/158) of anti-HCV+ (*p* < 0.001).

Awareness of current hepatitis infection was higher among individuals with HBsAg + compared to anti-HCV+ (*p* = 0.01). Awareness was 57.8% and 50.0% for HBV mono-infection, and HDV infection, respectively, in contrast, only approximately 10.2% of individuals with HCV (current or prior) infection were aware of their infection. Approximately 20–30% of participants with viral hepatitis were aware of infected family members. Overall, mean BMI was similar across groups (*p* = 0.73), but obesity was more prevalent among individuals anti-HCV + at 38.5% (60/156). Individuals with anti-HDV + had the highest median LS at 6.5 kPa (IQR 4.0-8.2) compared to other groups (*p* < 0.001). CAP scores were significantly lower in the anti-HDV + group (*p* = 0.04), and steatosis S3 was significantly more prevalent in individuals with HBV mono-infection at 30.8% (4/13) compared with 9.1% (1/11) in anti-HDV+, and 15.6% (10/64) in anti-HCV+ (*p* < 0.001).

### Prior screening of HBV infection

Only 19.3% (164/850) recalled prior viral hepatitis screening, with no sex difference (Table 1).

As shown in Supplementary Fig. 1a, prior HBV screening showed an increasing trend across years of migration, from 14.0% between the years 2000–2005, to 27.0% in years 2021–2023 (*p* = 0.037). Middle age groups (40–60 years) showed higher proportions of knowledge about prior screening compared with participants less than 30 and 30–40 years of age (Supplementary Fig. 1b; *p* = 0.003).

Overall, 15.5% (72/465) stated the presence of a prior or current infected family member, reported significantly more often by women than men (18.3% vs. 10.9% respectively, *p* < 0.001), while 39.1% regardless of sex were unaware of this information. For those with a positive family history (15.5%, 72/465), 68.1% (49/72) reported that a parent was infected, 38.9% (28/72) reported a sibling, and 5.6% (4/72) reported a child. Younger age groups (< 30, 30–40 years of age) had a lower prevalence of infected family members compared with older age groups, with overall 35–55% unawareness across all age groups, lowest in those aged 40-<50 years of age (Supplementary Fig. 2).

Overall, 96.4% (819/850) of individuals had successful liver elastography. the median value was 4.5 (IQR 3.7–5.6) kPa, with men showing higher values than women (4.8 vs. 4.3 kPa, *p* = 0.002). This difference remained significant across age groups 30–39, 40–49 and 50–59 years. For CAP scores, 31.3% (42/134) of men had S3 scores compared with 16.7% (34/204) of women (*p* < 0.001). Similarly, men had a more prevalent BMI ≥ 27.5 at 42.8% vs. 29.8% in women (*p* < 0.001).

### Follow-up and linkage to care

None of the 19 individuals with HBV mono-infection were eligible for anti-HBV therapy. Among individuals with anti-HDV + and HDV-RNA+, 18.2% (2/11) started Bulevirtide, 18.2% (2/11) started Tenofovir therapy, and 27.3% (3/11) patients initiated HCC surveillance (two women aged 50–59, and one man in his fifth decade). Two patients with anti-HDV + underwent additional procedures (one gastroscopy with non-selective beta-blocker prescription, one started anti-tuberculosis therapy). All patients with chronic HCV (*n* = 20) were offered DAA for 12 weeks and achieved sustained virological response in all recipients (100%) at follow-up.

### Prevalence of HBV, HDV in a comparator cohort in Mongolia

Among 413 randomly selected individuals screened for general health checkups at primary care clinics, 408 had available samples (mean age 41.3 ± 8.5 years; men constituted 43.6%, 178/408) (Supplementary Table 2). The prevalence of HBsAg + and anti-HDV + was 9.8% (40/408) and 5.1% (21/408), respectively. Men were younger and had a more prevalent HBsAg + than women (13.5% vs. 7.0%, *p* = 0.028). Among individuals with anti-HDV+, 78.9% (15/21) had HDV-RNA replication at a median log_10_ 5.9 (IQR 5.2–6.6) IU/mL. The mean ages of individuals with HBsAg+/anti-HDV-, HBsAg+/anti-HDV+, and those with HDV-RNA + were 39.8 (± 6.3), 44.2 (± 8.3) and 42.3 (± 8.4) years, respectively.

Compared with the Stockholm cohort, the Mongol general population cohort was slightly younger (41.3 vs. 43.1 years, *p* < 0.001), with a numerically higher male proportion (43.6% vs. 38.1%, *p* = 0.06). The prevalence of HBsAg+, anti-HDV+, HDV-RNA + was 9.8%, 5.1% and 3.7%, respectively, which were significantly higher than in the Stockholm screened cohort with corresponding 4.4%, 2.1% and 1.3% (all *p* < 0.05). Compared with the Stockholm cohort, the Mongol general population cohort had 2.39-fold higher odds of HBV infection (95% CI 1.50–3.80), 2.51-fold higher odds of anti-HDV+ (1.32–4.76), and 2.91-fold higher odds of HDV-RNA+ (1.33–6.39). (not shown in Table)

### Association between baseline characteristics with elevated LS ≥ 7.5 kPa (≥ F2 fibrosis)

In univariable analysis, older age, and any viral hepatitis were significantly associated with higher odds of elevated LS. In multivariable analyses, adjusting for age, sex, BMI, HDV-RNA and HCV-RNA replication, older age (adjusted OR [aOR] = 1.07, 95% CI 1.02–1.11, *p* = 0.002), HDV-RNA replication (aOR = 25.16, 95% CI 7.17–88.28, *p* < 0.001), and HCV-RNA replication (aOR = 7.20, 95% CI 2.41–21.47, *p* < 0.001) remained significantly associated with LS ≥ 7.5 kPa (Supplementary Table 3).

## Discussion

This first population-based screening of Mongolia-born individuals in Sweden revealed that the prevalence of chronic viral hepatitis B, D and C was 4.4%, 2.1% and 18.6%, respectively. The prevalence was significantly lower among those born after 1990. Only 8.7% had detectable anti-HBs, while ~ 19% reported prior HBV screening. Younger individuals (born after 1990) showed a low HBV vaccine protection rate. Most individuals with chronic viral hepatitis were unaware of their infection status, with a higher prevalence of advanced liver disease among those with HDV-RNA + compared with HBV mono- and HCV infection. This screening initiative linked 58 (6.8%) individuals to care.

The prevalence of viral hepatitis B, and D in Mongol migrants in Sweden was lower than Mongol national estimates [11, 13] but higher than Swedish general population estimates [8]. In the matched Ulaanbaatar cohort, we detected higher prevalences, reaching 9.8% for HBsAg + and 5.1% for anti-HDV+, consistent with published Mongol general population surveys [11, 24, 25]. Compared with other screening initiatives among Mongols living abroad, the 4.4% HBV prevalence in our study was lower than the 6.8%, 6.2% and 9.9% estimates reported in U.S. events in 2012, 2016 and 2021, respectively [14, 26, 27]. However, our estimated prevalence was comparable to the 3.6% HBV prevalence in a recent community-based screening of 222 Mongols across three Spanish cities [15]. The anti-HDV + prevalence of 48.6% among HBsAg + individuals in our study was higher than that reported in U.S. (41.2%) [14], and Spanish (0.9%) [15] screenings. Additionally, the prevalence of previous HCV infection in the present study (18.6%) was higher than in U.S [14]. and Spanish [15] events (9–10% and 5.8%, respectively). These variations may reflect differences in sex, age distribution, and time of enrollment across these cohorts [14, 15, 26, 27]. Notably, we observed a strong birth cohort effect for HCV infection, with higher prevalence among those born between 1945 and 1970, which aligns with prior Mongol studies [11]. The high prevalence of resolved HCV infection (86.7%) suggests prior linkage to care, possibly fewer reinfections, and improved survival in the middle-aged cohort. Although the prevalence of chronic HDV (62% with HDV-RNA replication) is consistent with published Mongol estimates [14], the lower prevalence compared with the matched cohort may suggest a healthy migrant effect [28]. Furthermore, the low prevalence of HBV, HCV, and absence of HDV infection in the younger birth cohort agrees with recent Mongol screenings [13].

Mongolia included HBV vaccination in the routine immunization schedule for neonates and children less than one year in 1991, with estimates of 99% of newborns receiving the first dose within 24 h of birth [29]. The Ministry of Health and the National Center for Communicable Diseases Mongolia, with WHO and UNICEF support, implemented a project to improve hepatitis B vaccination in 2005–2006 [29]. Timely administration of HBV vaccines and cold chain storage issues have been mentioned as possible factors affecting the quality assurance of HBV vaccine coverage and potency in Mongolia [13].

Studies have reported declining anti-HBs titers with age, regardless of region of origin [30, 31]. This waning effect of anti-HBs titers was also reported by Mongol researchers; Ochirbat and colleagues showed that only 10% of 438 children aged 5–10 years carried a protective anti-HBs titer [32], while others suggested an overall 41% protection [13]. Dashdorj and colleagues reported that anti-HBs titers without prior infection were detected in 33% of 3,800 children and young adults, declining with increasing age [33]. Our findings of low detected anti-HBs (16.4%) in the youngest cohort align with these reports. Studies have shown that long-term immunity may persist despite undetectable anti-HBs; subjects vaccinated early in life retain immune memory to HBV infection decades later regardless of anti-HBs detection [34]. As vaccination history is lacking in our study, along with other factors that might affect HBV vaccine responsiveness and access in Mongol migrants in Sweden; we could not further interpret these figures.

In our data, women comprised the majority of anti-HCV + and anti-HDV + cases, though proportionally lower than the HBV mono-infection group (not reaching statistical difference). This contrasts with the comparator Mongol cohort where men had 2.1-fold higher odds of HBV mono-infection compared with women (95% CI 1.07–4.06, *p* = 0.03; refer to Supplementary Table 2 for proportions). The cause of this disparity remains unclear. Nevertheless, although women had higher serological evidence of HCV and HDV infection in our analysis, they showed a trend towards fewer chronic infections (HCV-RNA and HDV-RNA replication, respectively). Moreover, women had significantly more prior HBV screening compared with men, possibly due to more antenatal screening, particularly for women in the reproductive age group, and higher health-seeking behavior compared with men, and women not in this age group or lacking such indication.

Unawareness of infection was high, reaching 50% in our analysis, mirroring similar figures from recent Mongol screening in other European countries [15], with greater unawareness for HDV. In Mongolia, 99% of individuals with HDV infection lacked knowledge of their infection, contrasted with 60% for HBV and HCV infection [13]. Nevertheless, the lack of screening in 70% in the < 40 years age groups highlights the need for targeted screening in populations from highly endemic regions, when earlier intervention might affect the course of disease progression, and survival. An encouraging finding in our study was that HBV screening increased from 14.0% (2000–2005) to 27.0% (2021–2023). Hur and colleagues reported 2.12-fold higher odds of completing three HBV vaccination doses associated with > 10 years of living in the United States [27]. In the current study, we could not identify an association between duration of stay in Sweden and the prevalence of HBV, HDV, and HCV infections. However, we assume that the low rate of chronic hepatitis C (13.4%) may reflect prior care and lack of repeated exposure.

Currently, liver societies recommend screening of persons from highly endemic regions for viral hepatitis (> 2% prevalence), while local guidelines implement different actions according to cost-effectiveness [35]. Although significant improvements have been achieved in timely diagnosis of viral hepatitis, delayed diagnosis combined with low awareness of infection have been associated with liver-related morbidity, especially among patients with chronic hepatitis B in high-income settings [10, 36]. Targeted screening might help assess and overcome gaps and disparities in the viral hepatitis cascade of care, though cost-effectiveness and stigma concerns remain. Rapid point-of-care tests could offer simple, easy-to-implement, and interpretable tests that enable faster diagnosis and immediate clinical decisions, possibly reducing loss to follow-up and improving linkage to care [37]. Recently, Dashdorj and colleagues developed and validated an anti-HDV antibody rapid diagnostic test that utilizes a defect-free bi-layer lipid membrane, which enhances biofouling resistance, minimizing non-specific binding, and significantly increases the sensitivity of molecular detection [38]. These tests have shown promising results for micro-elimination programs and in difficult-to-reach populations such as people who inject drugs and other vulnerable populations. Whether the use of home-based screening kits could further enhance linkage to care merits additional real-world studies.

Our study has several limitations. We did not explore factors such as education or area of residence that might affect not only the transmission of viral hepatitis but also the awareness and acceptance of health care. However, 70% of survey responders attended the event, reflecting good engagement. The study lacked information on vaccination status among participants; therefore, we relied on detection of anti-HBs+, acknowledging that long-term immunity might not necessarily correlate with anti-HBs titers [21]. Nevertheless, we sought serological evidence of detected anti-HBs regardless of quantitative level and presence of anti-HBc negative per WHO definition [20, 21]. Elevated LS predicted viral hepatitis infection in our model, but other confounders such as alcohol overconsumption and metabolic dysfunction were not assessed. Elevated alanine aminotransferase levels or laboratory-based scores such as FIB-4 (fibrosis index score) or APRI (aspartate-to-platelet ratio index) might be more feasible for detecting early liver diseases in healthy individuals [39]. Additionally, our finding of increased prevalence of overweight and obesity among study participants, particularly men, should be considered for future screening of metabolic-related morbidities in this population. We believe that this screening included estimated 10–20% of the Mongol population living in Sweden; however, selection bias favoring individuals who use social media and have internet access (extent unknown in this population) cannot be ruled out. Similarly, those who are aware of their disease status (even if not in care) might be less likely to attend such events. It is unclear whether the probability of screening and early diagnosis is higher in Sweden than in Mongolia. Given that such proactive population-screening efforts might be less available in countries with constrained resources, this highlights the importance of funding governmental and non-governmental organizations and initiatives in resource-limited settings.

## Conclusion

Our study revealed a higher prevalence of HBV and HDV infections among Mongols living in Stockholm than in Swedish general population estimates. The low rate of awareness, especially in younger age groups, calls for more tailored, age-specific efforts to increase awareness and engage this population. Nevertheless, the low level of vaccination protection, especially among the youngest birth cohort, merits further actions.

## Supplementary Information

Below is the link to the electronic supplementary material.

Supplementary Material 1

## Data Availability

All data were anonymized during data handling and analysis. The authors do not have permission to share the data.
