# Supplementary material for: Prevalence of chronic viral hepatitis B, D among Mongol migrants in Sweden compared to a sex- and age-matched native Mongol cohort
Source: BMC Infect Dis. 2026 Jan 16;26:178. doi: 10.1186/s12879-025-12506-w (PMC12849161; doi:10.1186/s12879-025-12506-w)
Supplement: Supplementary file 1 — Supplementary Material 1 [file 12879_2025_12506_MOESM1_ESM.docx]

**Prevalence of Chronic Viral Hepatitis B, D Among Mongol Migrants in Sweden Compared to a Sex- and Age-matched native Mongol Cohort**

Delgersaikhan Zulkhuu^1,2*^, Habiba Kamal^3,4,5*^, Sanjaasuren Enkhtaivan^1,2^, Ochirmaa Narantsogt^1,2^, Ganbolor Jargalsaikhan^1,2^, Munguntsetseg Batkhuu^1,2^, Byambasuren Ochirsum^1,2^, Marcus Ahl^3,4^, Karin Lindahl^3,4^, Michael Ingre^4,6^, Natalie Stiglund^7,8^, Alexandros Petropoulos^7,9^, Gustaf Sandh^7,10^, Niklas K. Björkström^7,8^, Sonja Saario^3^, Susanne Cederberg^3^, Annika Olsson^3^, Anna Wange Baye^3^, Ihssan Derdabi^3^, Desideria Georgos^3^, Andreas S. Bungert^1,2,11^, Nara Bungert Dashdorj^1,2,11^, ND Dashdorj Onom^1,2,11#^, Soo Aleman^3,4,5#^

*Shared first authorship

#shared senior authorship

Affiliations

1. The Liver Center, Ulaanbaatar, Mongolia
2. Onom Foundation, Ulaanbaatar, Mongolia
3. Department of Infectious Diseases, Karolinska University Hospital, Stockholm, Sweden
4. Department of Medicine Huddinge, Karolinska Institutet, Stockholm, Sweden
5. D-SOLVE Consortium, an EU Horizon Europe Funded Project, Hanover, Germany
6. Centre for Bioinformatics and Biostatistics, Karolinska Institutet, Stockholm, Sweden
7. Department of Clinical Microbiology, Medical Diagnostics Karolinska, Karolinska University Hospital, Stockholm, Sweden
8. Center for Infectious Medicine, Department of Medicine Huddinge, Karolinska Institutet, Karolinska University Hospital, Stockholm, Sweden.
9. Department of Microbiology, Tumor and Cell Biology, Karolinska Institutet, Stockholm, Sweden.
10. Division of Clinical Microbiology, Department of Laboratory Medicine, Karolinska Institutet, Stockholm, Sweden
11. Onom Institute, San Jose, California, United States

**Tables:**

Supplementary

Table 1: Parameters requested to be filled out by applicants to the online survey.

Table 2: Baseline characteristics of matched participants from a screening event in Mongolia conducted in Dec 2017-Jan 2018, sub-grouped by sex.

Table 3: Univariable and multivariable logistic regression model testing the association between participants characteristics and liver stiffness measurements ≥7.5 kPa. Crude (OR) and adjusted odds ratio (aOR) are presented with 95% CI.

**Figures:**

Supplementary

Figure 1: Knowledge on prior HBV testing by a) year of immigration and b) by age categories.

Figure 2: Knowledge on prior or current infection in a family member in participants by age categories.

| **Table 1: Parameters requested to be filled by applicants to the online survey*** |
| --- |
| Email |
| Name |
| Family name |
| First name |
| Mongolian registration number Регистрийн дугаар |
| E-mail address E-майл хаяг |
| Birth date Төрсөн он сар өдөр |
| Mobile phone number |
| Attended (yes, no) |
| Additional contact phone number Нэмэлт утасны дугаар |
| Which day would you like to come in for testing during “Find the Missing Thousands Campaign” - Stockholm?  Элэгний вирүсээ илрүүлье, эмчилье - Стокхолм арга хэмжээ Швед Улсын Стокхолм хотод зохион б... |
| Do you have personal identification number (Swedish personal number)? Танд Швед Улсын хувь хүний ID дугаар байгаа эсэх? |
| If yes, please write here your Swedish personal number! Тийм бол Швед Улсын хувь хүний ID дугаараа бичнэ үү! |
| If you do not have Swedish personal number but do you have LMA number given by Migrationsverket (Swedish Migration Agency)?  Хэрэв танд Швед Улсын хувь хүний ID дугаар байхгүй, гэхдээ Швед Улсын ш... |
| If yes, please state here! Тийм бол LMA дугаараа бичнэ үү! |
| Sex Хүйс |
| Height [cm] Өндөр [см] |
| Weight [kg] Жин [кг] |
| How many years have you now been outside Mongolia? Та хэдэн жилийн хугацаанд Монголоос гадна гадаад улсад амьдарч байна вэ? |
| What year did you come to Sweden? Та хэдэн онд Швед Улсад ирсэн бэ? |
| Have you been tested for hepatitis B before (please choose the right answer)? Та өмнө нь хепатитын В вирүсийн шинжилгээ өгч байсан уу (зөв харилтыг сонгоно уу)? |
| If yes on previous question, have you been diagnosed with hepatitis B previously?  Хэрэв асуултын хариулт тийм бол, та өмнө В вирүст хепатиттай гэж оношлогдож байсан уу? |
| If yes on previous question, were you diagnosed with hepatitis D previously? Хэрэв асуултын хариулт тийм бол, та өмнө нь Дельта вирүст хепатиттай гэж оношлогдож байсан уу? |
| *Do you have any close family member with diagnosis of hepatitis B or D (please choose the right answer)? Таны гэр бүлийн гишүүдэд хепатитын В эсвэл Дельта вирүсийн халдвартай гэж оношлогдож байсан... |
| If yes on *question, was it a parent? Хэрэв * дугаар асуултын хариу тийм бол, энэ оношлогдсон хүн нь таны эцэг эх байсан уу? |
| If yes on previous question, do you know which diagnosis? Хэрэв тийм бол, та оношийг аль нь гэдгийг мэдэх үү? |
| If yes on *question, was it a sibling? Хэрэв *дугаар асуултын хариу тийм бол, энэ оношлогдсон хүн нь таны ах, эгч, эсвэл дүү байсан уу? |
| If yes on previous question, do you know which diagnosis? Хэрэв тийм бол, та оношийг аль нь гэдгийг мэдэх үү?2 |
| If yes on *question, was it your child?  Хэрэв * дугаар асуултын хариу тийм бол, энэ оношлогдсон хүн нь таны хүүхэд байсан уу? |
| If yes on previous question, do you know which diagnosis?  Хэрэв тийм бол, та оношийг аль нь гэдгийг мэдэх үү? |
| Abbreviations: LMA=card granted for asylum seekers in Swedish ”*Lagen om mottagande av asylsökande”.* Questions* are provided in Mongolian with English translation. |

| **Table 2: Baseline characteristics of matched participants from a screening event in Mongolia conducted in Dec 2017-Jan 2018, sub-grouped by sex** | | | | |
| --- | --- | --- | --- | --- |
| **Parameters, n (%) unless stated otherwise** | All | Men | Women | p-value |
| **Total number** | 408 | 178 (43.6) | 230 (56.4) | <0.001 |
| Age, mean (sd) | 41.3 (8.5) | 39.9 (8.7) | 42.4 (8.3) | 0.009 |
| HBsAg positive | 40 (9.8) | 24 (13.5) | 16 (7.0) | 0.028 |
| HBsAg log_10_ (IU/ml), median (IQR) | 3.4 (2.4-3.8) | 3.4 (2.6-3.7) | 3.3 (2.3-4.2) | 0.97 |
| **HDV** |  |  |  |  |
| Anti-HDV+ | 21 (5.1) | 11 (6.2) | 10 (4.3) | 0.30 |
| HDV-RNA+** | 15 (78.9) | 7 (77.8) | 8 (80.0) | 0.18 |
| HDV RNA log10 IU/ml, median (IQR) | 5.9 (5.2-6.6) | 6.0 (5.2-6.9) | 5.6 (4.9-6.2) | 0.25 |
| *Abbreviations: sd= standard deviation; anti-HDV= hepatitis D antibody; HDV RNA= hepatitis D virus ribonucleic acid;***available samples to quantify (n=19, 9 men/10 women). | | | | |

| **Table 3: Univariable and multivariable logistic regression model testing the association between all participants characteristics and liver stiffness measurements ≥7.5 kPa. Crude (OR) and adjusted odds ratio (aOR) are presented with 95% CI.** | | | | |
| --- | --- | --- | --- | --- |
|  | **Univariable**  **OR (95% CI)** | **p-value** | **Multivariable***  **aOR (95% CI)** | **p-value** |
| Age at the event | **1.07 (1.03-1.10)** | **<0.001** | **1.07 (1.02-1.11)** | **0.002** |
| Sex (women as reference) | 1.00 (0.54-1.87) | 0.99 | 1.10 (0.56-2.14) | 0.79 |
| Length of stay in Sweden per year | 0.99 (0.93-1.06) | 0.77 |  |  |
| Positive family history for HBV | 1.81 (0.63-5.18) | 0.27 |  |  |
| BMI (continuous scale) | 1.06 (1.00-1.12) | 0.06 |  |  |
| HBsAg+ | **4.66 (1.92-11.31)** | **<0.001** |  |  |
| Anti-HDV + | **9.96 (3.55-27.98)** | **<0.001** |  |  |
| HDV-RNA+ | **24.13 (7.05-82.60)** | **<0.001** | **25.16 (7.17-88.28)** | **<0.001** |
| Anti-HCV+ | **2.45 (1.28-4.70)** | **0.007** |  |  |
| HCV RNA+ | **6.44 (2.23-18.64)** | **<0.001** | **7.20 (2.41-21.47)** | **<0.001** |
| Any viral hepatitis | **3.37 (1.82-6.24)** | **<0.001** |  |  |
| *Abbreviations: *Sex, age, BMI, HDV RNA+ and HCV RNA+ are introduced in the model. HBsAg= hepatitis B surface antigen; OR=odds ratio; BMI= body mass index; CI=confidence interval.* | | | | |

Supplementary figures

Figure 1: Knowledge on prior HBV testing by a) year of immigration and b) by age categories.

**a**

**b**

Figure 2: Knowledge on prior or current infection in a family member in participants by age categories

Number of responders to this question are shown in brackets.
